# Characterization and Proteomic Analyses of Proinflammatory Cytokines in a Mouse Model of Liver Transplant Rejection

**DOI:** 10.1155/2022/5188584

**Published:** 2022-08-12

**Authors:** Shi-Peng Li, Xin-Qiang Li, Xiao-Jie Chen, Jin-Ming Zhang, Guang-Peng Zhou, Liu-Xin Zhou, Hai-Ming Zhang, Li-Ying Sun, Zhi-Jun Zhu

**Affiliations:** ^1^Liver Transplantation Center, Beijing Friendship Hospital, Capital Medical University, Beijing 100050, China; ^2^Clinical Research Center for Pediatric Liver Transplantation of Capital Medical University, Beijing 100050, China; ^3^National Clinical Research Center for Digestive Diseases, Beijing 100050, China; ^4^Organ Transplantation Center, Affiliated Hospital of Qingdao University, Qingdao 266000, China; ^5^Department of Critical Liver Disease, Beijing Friendship Hospital, Capital Medical University, Beijing 100050, China

## Abstract

Liver transplantation (LT) is an effective strategy for the treatment of end-stage liver disease, but immune rejection remains a significant detriment to the survival and prognosis of these LT patients. While immune rejection is closely related to cytokines, the cytokines investigated within previous studies have been limited and have not included a systematic analysis of proinflammatory cytokines. In the present study, we used a protein chip system and proteomics to detect and analyze serum proinflammatory cytokines and differentially expressed proteins in liver tissue in a mouse model of liver transplantation. In addition, bioinformatics analysis was employed to analyze the proinflammatory cytokines and differential changes in proteins in response to this procedure. With these analyses, we found that serum contents of GC-CSF, CXCL-1, MCP-5, and CXCL-2 were significantly increased after liver transplantation, while IL-5, IL-10, and IL-17 were significantly decreased. Results from Gene Ontology (GO) and KEGG pathway analyses revealed that the cytokine-cytokine receptor, Th1/Th2 cell differentiation, and JAK-STAT signaling pathway were enriched in a network associated with the activation of immune response. Results from our proteomic analysis of liver tissue samples revealed that 470 proteins are increased and 50 decreased, including Anxa1, Anxa2, Acsl4, Sirpa, S100a8, and S100a9. KEGG pathway analysis indicated that the neutrophil extracellular trap formation, NOD-like receptor signaling pathway, and leukocyte transendothelial migration were all associated with liver transplant rejection in these mice. Bioinformatics analysis results demonstrated that CXCL-1/CXCL-2 and S100a8/S100a9 were the genes most closely related to the functions of neutrophils and the mononuclear phagocyte system. These findings provide new insights into some of the critical factors associated with liver transplant rejection and thus offer new targets for the treatment and prevention of this condition.

## 1. Introduction

It is well established that liver transplantation is the most effective surgical procedure for the treatment of end-stage liver disease [1]. However, there remain many problematic issues affecting the survival and prognosis of these patients [2], with immune rejection being one of the most notable [3]. Following liver transplantation, a substantial number of recipient immune cells (neutrophils, lymphocytes, macrophages, monocytes, and dendritic cells) infiltrate the donor liver to create a new immune environment [4]. It is these immune cells that determine the occurrence and development of liver transplant rejection.

The mechanisms underlying liver transplant rejection have received considerable attention lately [5, 6], with these studies mainly focusing on cellular and humoral immunity, as well as innate immunity responses as related to liver transplant rejection [7–9]. Dendritic cells, monocytes, macrophages, and neutrophils have also all been shown to play a vital role in regulating innate immunity [10, 11]. The occurrence of immune rejection is closely related to chemokines, proinflammatory cytokines, and signaling pathways [12, 13]. As cytokines represent important mediators of the immune system, they may serve as potential therapeutic agents which could selectively inhibit or enhance immune responses [14]. In support of this relationship among cytokines, the immune system and transplantation are the findings that proinflammatory cytokines (G-CSF, CXCL-2, and CXCL-1) and lymphocyte activating cytokines (IL-2, IL-4, and IL-6) are increased during ex vivo human lung perfusion [15]. Moreover, donor-reactive CD8^+^ T cells utilize the CXCR-3 chemokine axis as a costimulatory pathway during priming to allografts while CXCL-9 promotes the development of IFN-g-producing CD8^+^ T cells [16]. Interestingly, levels of IL-2 and IL-6 in stimulated peripheral lymphocyte supernatants of kidney transplant recipients can predict acute renal allograft rejection [17]. IL-10-producing ILC2 cells can lead to long-term survival of islet grafts, suggesting that these IL-10-producing ILC2 cells are needed to obtain a maximal protective effect on these allografts [18]. However, these studies were all limited to investigating the effects of chemokines and did not include any systematic analyses of proinflammatory cytokines. Accordingly, the potential role of proinflammatory cytokines in liver transplant rejection remains unknown.

In this study, we used an antibody array assay (protein chip system), along with proteomics (isobaric tags for relative and absolute quantification (iTRAQ)) to detect and analyze potential changes in serum proinflammatory cytokines and proteomics within liver tissue in a mouse model of liver transplantation. Moreover, with the use of bioinformatics analysis, we analyzed the changes in proinflammatory cytokines and proteins within liver tissue of these mice. With these analyses, we were able to identify functional proteins that were related to correlations between proinflammatory cytokines and transplant rejection. Such findings can serve as the foundation for the development of novel therapeutic targets for the prevention and treatment of liver transplant rejection.

## 2. Materials and Methods

### 2.1. Animals

Male C57BL/6J mice (*n* = 9) were used as donors and male C3H/He mice (*n* = 6) as recipients. All mice were 8-10 weeks old (BW = 23 ± 2 g) and purchased from SiPeiFu, Beijing, Biotechnology Co., LTD). The mice were maintained in a specific pathogen-free (SPF) environment and housed in accordance with laboratory animal care principles.

### 2.2. Mouse Orthotopic Liver Transplantation (OLT) Model

**Figure 1:**
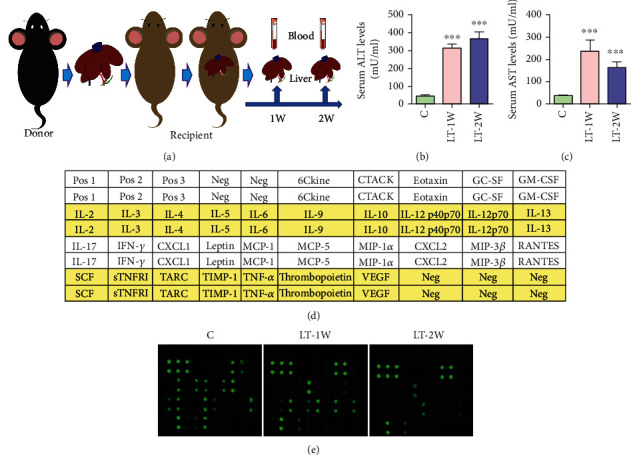
Mouse orthotopic liver transplantation model. (a) Pattern diagram of mouse orthotopic liver transplantation model. (b, c) Serum levels of ALT and AST following liver transplantation. (d) Inflammation antibody array G-series 2 map including 32 cytokines. (e) Cytokine levels were proportional to their fluorescent intensities. ^∗^*P* < 0.05,  ^∗∗^*P* < 0.01, and^∗∗∗^*P* < 0.001 compared with the control group.

Orthotopic liver transplantation surgeries were performed while mice were under isoflurane inhalation anesthesia, according to procedures described previously [19]. All mice were divided into three groups (*n* = 3/group): liver transplant one week (LT-1W), liver transplant two weeks (LT-2W), or control group (C), with the 1W and 2W designations referring to the times (weeks) posttransplant when determinations were performed (Figures 1(a)). Three mice per group were harvested for blood and liver samples in LT mice.

### 2.3. Serum AST and ALT Determinations

Blood samples from all mice were collected, and serum was obtained following centrifugation. Serum AST and ALT levels were determined using the mouse transaminase test kit (Shanghai Yaji Biotechnology, China) [20].

### 2.4. Histopathology of Liver Samples

Fresh liver samples from each group were fixed in 4% paraformaldehyde solution for 24 h, then dehydrated and embedded in paraffin. Sections (4 *μ*m) were cut from these paraffin-embedded tissue samples and stained with HE or Masson to evaluate the degree of liver injury.

### 2.5. Immunocytochemistry

Paraffin sections were deparaffinized and rehydrated following routine methods. Antibodies against CD14 (Abcam, Cambridge, MA, USA) and CD11b (Abcam, Cambridge, MA, USA) were initially incubated at 4°C overnight with secondary antibodies being incubated for 1 h at 37°C after three washings with staining buffer. Diaminobenzidine staining was performed using a diaminobenzidine peroxidase substrate kit (ZSGB-BIO, Beijing, China). The slides were observed under light microscopy.

### 2.6. Protein Chip System for Serum

An aliquot from serum samples was shredded using mouse inflammation array G-Series 2 (Raybiotech, Norcross, GA, United States) to obtain a protein extract. With this analysis, 32 different cytokines were detected according to the Raybiotech analysis tool. Signal values were read and then normalized [21].

### 2.7. Protein Extraction

Fresh liver tissue samples were minced into small fragments, washed with 1x PBS buffer, and then homogenized with lysis buffer. Total protein content was determined using tryptophan fluorescent analysis in the form of a microtitration plate. In addition, MEDFASP was used to continuously digest proteins using LysC, trypsin, and chymotrypsin to treat proteins in small samples containing 15 *μ*g of tissue.

### 2.8. LC-MS/MS Mass Spectrometry

In this study, we used the Q Exactive HF-X mass spectrometer and a Nanospray Flex™ (NSI) ion source, with the ion spray voltage set at 2.4 kV and ion transmission tube temperature at 275°C. A data-dependent acquisition mode was adopted for mass spectrometry. The parent ions with an ion strength of Top40 in the full scan were selected and broken using the high-energy collision cracking (HCD) procedure. These ions were detected using secondary mass spectrometry to generate the original mass spectrometry detection data (.raw).

### 2.9. Evaluation and Statistical Analysis of Identified Proteins

The distributions of peptide and PSM numbers of matching proteins were analyzed. Molecular weight distributions of identified proteins and protein coverage were determined based on the peptide.

### 2.10. Screening of Differentially Expressed Proteins

Relative protein quantitation was calculated as an average ratio with a change of >1.5-fold being considered statistically significant (*P* ≤ 0.05). Candidate proteins were examined using the Protein ID of the Protein Pilot software program.

### 2.11. Analysis and Annotation Functions

Functional annotation and analysis were used to extract various structural and functional annotation information from these molecules based on the current annotation database (Gene Ontology, KEGG) list of experimentally identified genes or other molecules.

### 2.12. Protein Interaction Analysis

The analysis of interactions between proteins in cells can reveal their function at the molecular level. This analysis focuses on the differentially expressed proteins of interest to investigators and constructs a network to identify the relationship between these differentially expressed proteins and the possible functional groups.

### 2.13. Statistical Analysis

Statistical comparisons were performed using one-way ANOVA to analyze differences among the groups. The SPSS software 20.0 program was used in these analyses, and a *P* < 0.05 was required for results to be considered statistically significant.

## 3. Results

### 3.1. Mouse Orthotopic Liver Transplantation (OLT) Model

A summary of changes in serum ALT and AST levels in these OLT mice is shown in Figures 1(b) and 1(c). As compared with that in the control group, levels of ALT and AST increased gradually in the LT-1W and LT-2W groups. In these OLT mice, serum ALT levels peaked at 2 weeks, while AST at 1 week after transplantation. Serum levels were also detected using the protein chip system, with Figure 1(d) illustrating the cytokine layout of the protein chip and Figure 1(e) the protein chip fluorescent picture.

### 3.2. Proinflammatory Cytokines in the OLT Mouse Model

**Figure 2:**
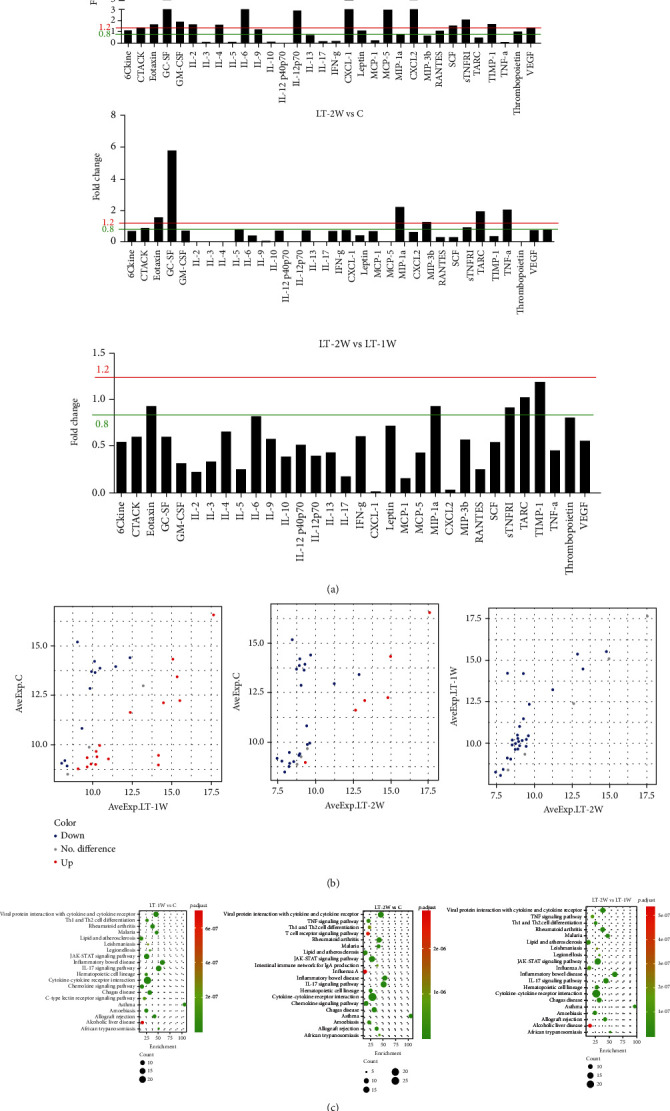
Serum levels of proinflammatory cytokines. (a) Differentially expressed proinflammatory cytokines of mice within each group. (b) Scatter plot of proinflammatory cytokines. (c) KEGG analysis of proinflammatory cytokine functions.

As shown in Figures 2(a) and 2(b), a number of cytokines were significantly increased (notably GC-CSF, GM-CSF, IL-12-p70, IL-2, IL-4, IL-6, CXCL-1, MCP-5, CXCL-2, sTNFRI, and TIMP-1) and a number significantly decreased (notably IL-3, IL-5, IL-10, IL-17, IFN-g, MCP-1, TARC, and TNF-a) in the LT-1W as compared with the control group. Within the LT-2W group, there were also increases in some cytokines (notably GC-CSF, MCP-5, CXCL-2, sTNFRI, and TIMP-1) and decreases in others (notably IL-2, IL-3, IL-4, IL-6, IL-10, IL-17, CXCL-1, IFN-g, MCP-1, TARC, and TNF-a) as compared with the control group. When comparing the LT-1W versus LT-2W groups, a number of cytokines (notably IL-2, IL-3, IL-4, IL-6, IL-10, IL-17, CXCL-1, CXCL-2, IFN-g, MCP-1, and TNF-a) were found to be significantly decreased in the LT-2W group. These results reveal that the proinflammatory cytokines, GC-CSF, GM-CSF, MCP-5, CXCL-1, and CXCL-2, which are closely related to monocytes, macrophages, and neutrophils showed significant, temporally dependent changes in response to liver transplant surgery. Bioinformatics analysis was also performed on these proinflammatory cytokines (Figure 2(c)), with the results that the cytokine-cytokine receptor, Th1/Th2 cell differentiation, JAK-STAT signaling pathway, cytokine-cytokine receptor interaction, and chemokine signaling pathway in the LT-1W group all showed significant changes as compared with the control group. Moreover, cytokine-cytokine receptor, TNF signaling pathway, Th1/Th2 cell differentiation, and T cell receptor signaling pathway changed obviously in the LT-1W group. Compared with the LT-1W group, the signal pathways showing obvious changes included the cytokine-cytokine receptor, TNF signaling pathway, Th1/Th2 cell differentiation, and T cell receptor signaling pathway in the LT-2W group. The significant changes in proinflammatory cytokines and pathways, as summarized in Figure 2, may be related to the marked changes in macrophages, monocytes, and neutrophils as observed after liver transplantation.

### 3.3. Alterations in Phagocytes in the OLT Mouse Model

**Figure 3:**
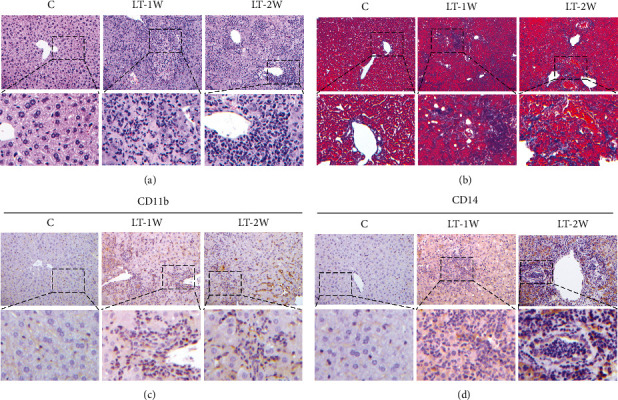
Immune cell infiltration within the liver graft. (a) HE and (b) Masson staining to evaluate the extent of liver damage after liver transplantation. (c, d) Immunocytochemistry for determination of CD11b and CD14 expression within the livers of each group.

In the LT-1W group, there were increases in inflammatory cell (neutrophils, monocytes, macrophages, and lymphocytes) infiltration within the liver, as well as enhanced congestion, swelling, and necrosis of hepatocytes as compared with the control group (Figure 3(a)). Compared with the LT-1W group, macrophages, monocytes, and neutrophils were relatively increased in the LT-2W, while lymphocytes were decreased in the LT-2W group. Liver tissue injury was also assessed using Masson staining to detect liver tissue fibrosis (Figure 3(b)). Compared with that observed in the control group, the degree of fibrosis gradually increased in LT groups. Results from immunohistochemical staining revealed few CD11b positive cells in normal liver tissue, while the number of these CD11b positive cells was increased within the liver of the LT-1W group and was further increased in the LT-2W group (Figure 3(c)). As can be seen from Figure 3(d), compared with the control group, the number of CD14 positive cells increased significantly in the LT-1W group. Meanwhile, CD14 positive cells also increased in the LT-2W group. While large numbers of CD11b and CD14 positive cells were found in the liver after liver transplantation, these CD11b or CD14 positive cells mainly existed in monocytes, macrophages, and neutrophils. Accordingly, these reveal that increases in monocytes, macrophages, and neutrophils present within the livers of these OLT mice and provide an assessment of the proteomics within this liver tissue.

### 3.4. Construction of a Protein Map in the OLT Mouse Model Using Proteomics

**Figure 4:**
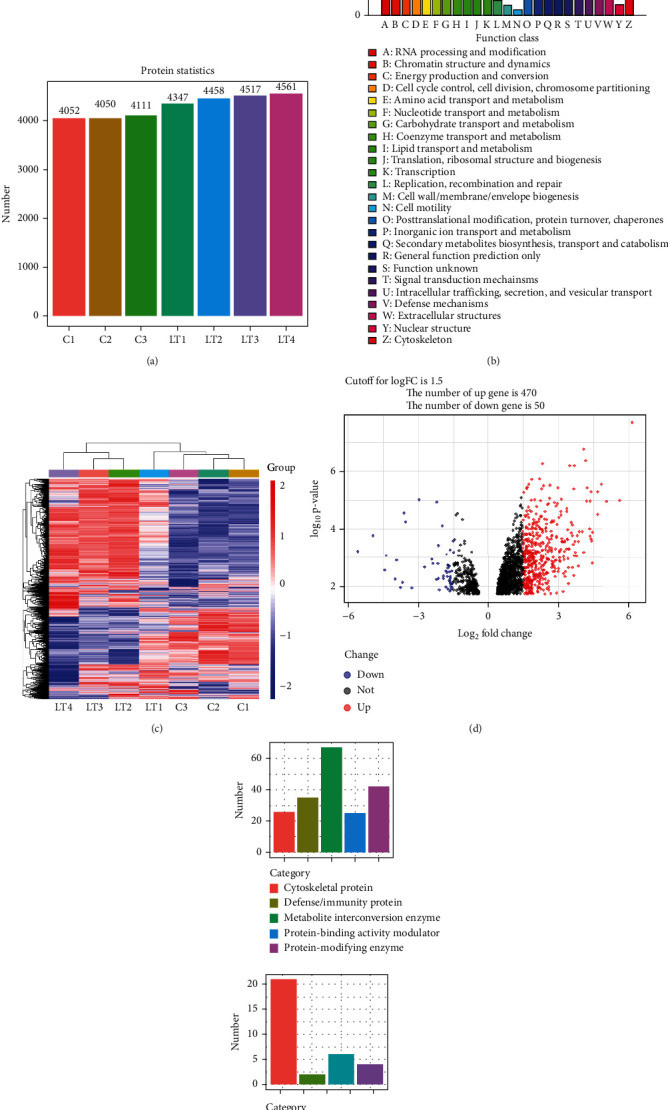
Proteomic results for determination of protein expression in liver tissue. (a) Number of proteins extracted from selected mouse liver tissue samples. (b) Number of proteins and COG functional classification in each mouse liver tissue sample. (c) Differences in proteins among the groups, in which 470 proteins were increased and 50 decreased (d). (e) The differential proteins obtained can be classified into five or four categories.

A summary of the number of proteins extracted from selected mouse liver samples is shown in Figure 4(a), while Figure 4(b) contains the number of proteins and COG functional classification in each of these liver samples. Overall, of the different proteins observed within these groups (Figure 4(c)), 470 proteins were increased and 50 proteins decreased in the LT group (Figure 4(d)). These differentially expressed proteins can be classified into either a five-category (cytoskeletal protein, defense/immunity protein, metadata conversion enzyme, protein binding activity modulator, and protein modifying enzyme) or a four-category (metadata conversion enzyme, protein modifying enzyme, transfer/carrier protein, and transporter) classification, with the number of proteins being different within each category (Figure 4(e)).

### 3.5. Pathway Enrichment Analysis of Differentially Expressed Proteins

**Figure 5:**
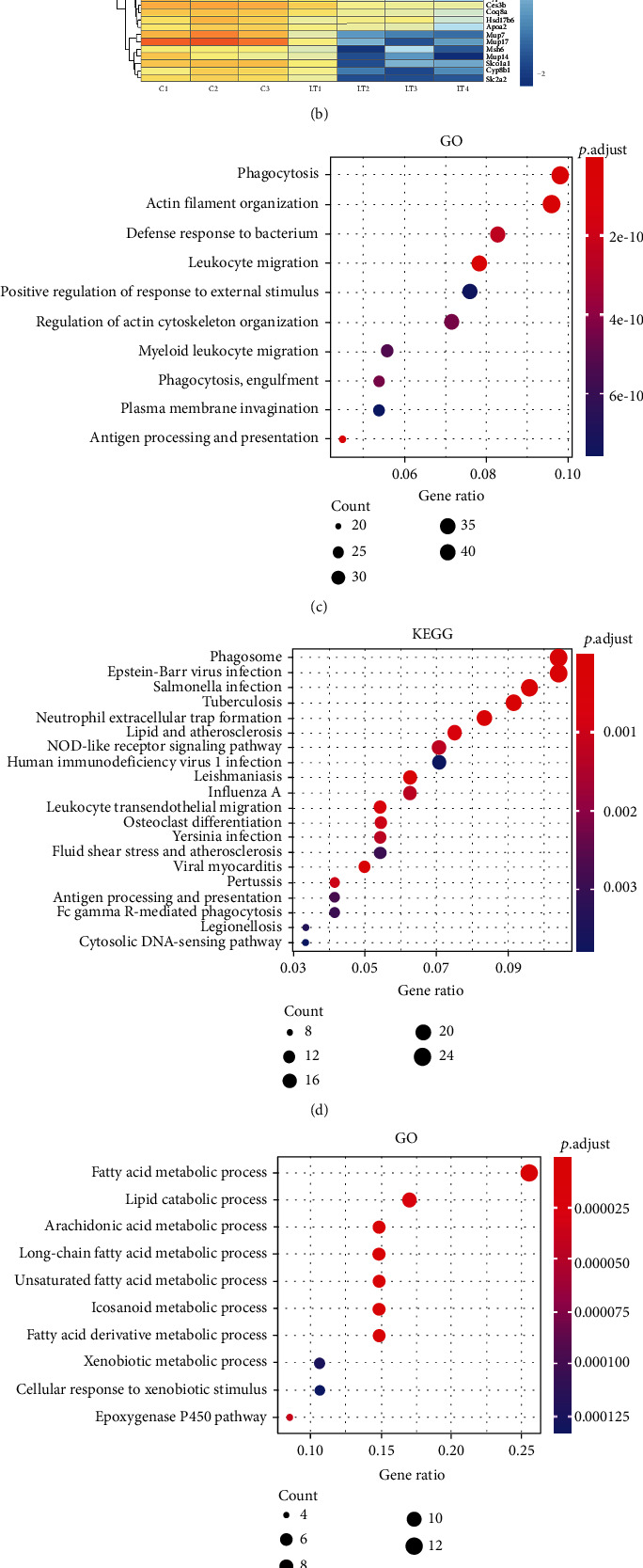
Analysis and annotation functions of differential proteins after liver transplantation. (a, b) Hierarchical cluster analysis of proteins. (c) GO analysis and (d) KEGG analysis of upregulated proteins. (e) GO analysis and (f) KEGG analysis of downregulated proteins.

A bioinformatics analysis was applied to evaluate the different proteins observed within the liver tissue of these mice. For this analysis, we selected the top 50 proteins with increased expression and top 50 proteins with decreased expression (Figures 5(a) and 5(b)). In addition, GO and KEGG pathway analyses were performed on those proteins showing increased or decreased expressions (Figures 5(c), 5(d), 5(e) and 5(f)). Results of the GO analysis indicated that the increased protein functions mainly involved phagocytosis, leukocyte migration, and myeloid leukocyte migration, while reduced protein expressions were mainly related to changes in fatty acid metabolic process, lipid catabolic process, and arachidonic acid metabolic processes. The KEGG pathway analysis showed that neutrophil extracellular trap formation, lipid and atherosclerosis, the NOD-like receptor signaling pathway, and leukocyte transendothelial migration were related to liver transplant rejection in these mice and that it is mainly related to steroid hormone biosynthesis, retinol metabolism, and chemical carcinogenesis-DNA adducts.

### 3.6. Protein-Protein Interaction Network Analysis

**Figure 6:**
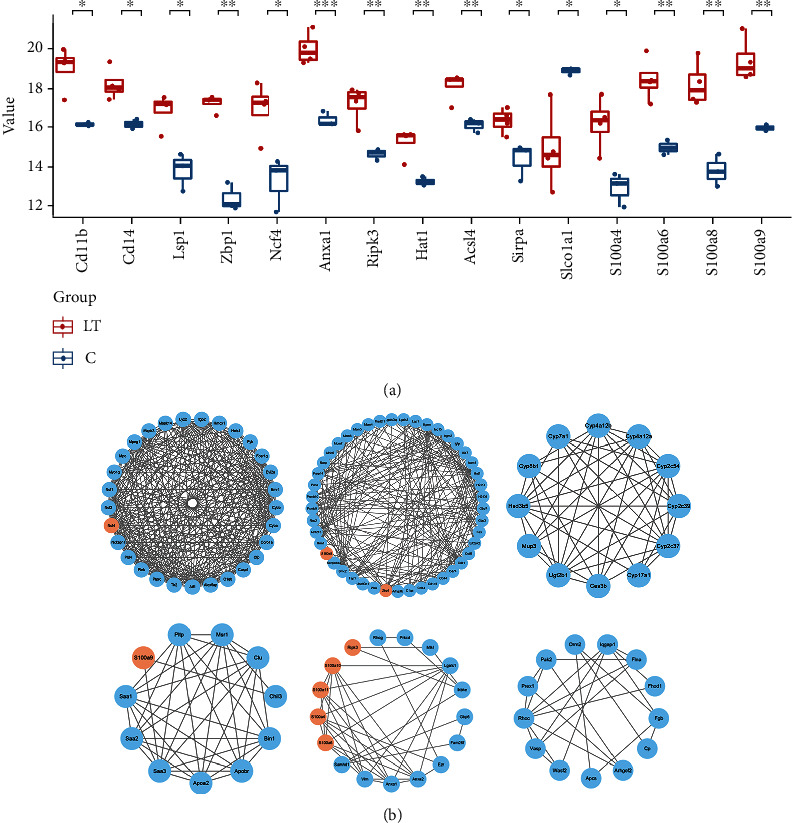
Protein interaction analyses of upregulated proteins after liver transplantation. (a) Selected highly expressed proteins were screened from the differential proteins obtained from liver transplantation. (b) Network analyses of differential nodes represent proteins and lines between the nodes indicating direct protein-protein interactions.

Proteins with increased expression, including CD11b, CD14, Lsp1, Zbp1, Ncf4, Ripk3, Acsl4, Sirpa, S100a1, S100a4, S100a6, S100a8, and S100a9 (Figure 6(a)), were screened, and interactions among these different proteins were then analyzed. As shown in Figure 6(b), S100a8 interacted with the CD44 protein, S100a9 with the Chil3 protein, and S100a10 with the Anxa1/Anxa2 protein. Results of the KEGG pathway analysis revealed that these proteins were closely related to the functions of monocyte macrophages and neutrophils.

## 4. Discussion

Following liver transplantation, a substantial number of immune cells enter the donor liver and interact with immune cells of the graft [22], an effect which leads to increases in immune responses and inflammatory reactions [23]. During this process, the liver will produce and release a large number of cytokines, including chemokines and proinflammatory cytokines. Cytokines, which can exert a wide range of effects upon proinflammatory and regulatory properties, might be considered potential therapeutic targets for selective suppression or enhancement of the immune responses in recipients [24]. In the mouse OLT model, we found that serum ALT and AST showed a temporally dependent increase after liver transplantation, indicating that hepatocytes were damaged. Liver injury after transplantation is often due to inflammatory reaction and rejection. With rejection, a large number of proinflammatory cytokines can recruit neutrophils, monocytes, and macrophages into the grafts and, in this way, contribute to inflammation and rejection.

Results of the mouse serum protein chip analysis indicated that a large number of proinflammatory cytokines, including GC-CSF, CXCL-1, MCP-5, and CXCL-2, were released one week after liver transplantation. Simultaneously, contents of lymphocyte activating cytokines, such as IL-5, IL-10, and IL-17, were decreased. About two weeks after operation, the contents of GC-CSF and MCP-5 were still high, while the contents of proinflammatory cytokines such as CXCL-1 and CXCL-2 decreased. In response to transplant rejection, cells in the liver can produce several proinflammatory chemokines and cytokines [25]. Among these cytokines, CXCL-1 and CXCL-2 serve as chemoattractants for neutrophils [26]. Results, as obtained from staining, also show that a large number of immune cells, including neutrophils, monocytes, macrophages, lymphocytes, and other immune cells, infiltrated the graft after liver transplantation. Therefore, in the early stages after liver transplantation, we observed substantial amounts of proinflammatory cytokines, within the serum in our OLT mouse model. Such an effect could then increase the number of innate immune cells participating in transplant rejection.

Proteomics were then used to identify changes in liver proteins associated with transplantation. With this analysis, we found that 470 proteins were increased and 50 decreased. The increased proteins were closely related to leukocyte metastasis and the NOD-like receptor pathway, while the decreased expression of proteins was mainly related to lipid metabolism and the PPAR pathway. Notably, the NOD-like receptor proteins NOD1 and NOD2 can participate in innate immune responses [27], and NOD1 stimulates the release of chemokines, such as CXCL-8, CXCL-1, and CXCL-2, which can attract neutrophils to the site of infection [28].

Further analysis of proteins revealed that significant increases were obtained in the expressions of CD11b, CD14, S100a4, S100a8, and S100a9, as well as in other proteins. These proteins are closely related to the functional activities of neutrophils, monocytes, and macrophages; in particular, CD11b, S100a8, and S100a9 form the heterodimer, calprotectin, which is released by activated monocytes/macrophages and neutrophils [29], and S100a8/S100a9 can also produce a macrophage-induced inflammation and degeneration of the vessel wall. Such effects may then provide an explanation for the increased levels of intra-aneurysmal S100a8 and S100a9 as observed in ruptured versus nonruptured intracranial aneurysms [30]. Results from our serological assays revealed that CXCL-1 and CXCL-2 were significantly increased, which could exert an important effect on the chemotaxis and functional activation of neutrophils and the mononuclear phagocytic system. In addition, our proteomic findings indicated that there was an increase in the content of S100a8/S100a9 within the liver, suggesting that a close relationship may exist between CXCL-1/CXCL-2 and S100a8/S100a9. From our analyses of protein-protein interactions, we found that S100a9 interacted with Saa3 and Chil3 and that S100a4/S100a6 were closely related to Anxa1/Anxa2. It has been reported that CXCL-1 and CXCL-2 are closely related to S100a8/S100a9. Anti-inflammatory proteins can combine to transcriptionally repress hepatic expression of S100a8, S100a9, CXCL-1, and CXCL-2 [31], and TLR2 and S100a8/S100a9 represent key regulators of hepatic CXCL-2 expression and neutrophil recruitment [32].

In summary, our current results suggest that increased secretion of CXCL-1 and CXCL-2 can enhance the functional activity of neutrophils and the mononuclear phagocyte system within the donor liver to then participate in processes leading to liver rejection. During this process, infiltrating neutrophils and monocyte/macrophages are associated with high expression of S100a8/S100a9, and calprotectin can further regulate the functions of these cells to contribute to the immune responses observed.

## Data Availability

All data generated or analyzed during this study are available in this article.

## Abbreviations

LT: Liver transplantation
AST: Aspartate aminotransferase
ALT: Alanine aminotransferase
TBil: Total bilirubin
IL: Interleukin
GO: Gene Ontology
CXCL: Chemokine (C-X-C motif) ligand
CCL: Chemokine (C-C motif) ligand
IFN: Interferon
KEGG: Kyoto Encyclopedia of Genes and Genomes
NOD: Nucleotide binding oligomerization domain containing
SPF: Specific pathogen-free
HE: Hematoxylin-eosin staining.
